# Dental Pulp Stem Cell Mechanoresponsiveness: Effects of Mechanical Stimuli on Dental Pulp Stem Cell Behavior

**DOI:** 10.3389/fphys.2018.01685

**Published:** 2018-11-26

**Authors:** Massimo Marrelli, Bruna Codispoti, Richard M. Shelton, Ben A. Scheven, Paul R. Cooper, Marco Tatullo, Francesco Paduano

**Affiliations:** ^1^Stem Cells Unit, Biomedical Section, Tecnologica Research Institute and Marrelli Health, Crotone, Italy; ^2^School of Dentistry, Institute of Clinical Sciences, College of Medical and Dental Sciences, The University of Birmingham, Birmingham, United Kingdom

**Keywords:** dental pulp stem cells (DPSCs), mechanobiology, mechanosensing, mechanical properties, behavior, surface topography

## Abstract

Dental pulp is known to be an accessible and important source of multipotent mesenchymal progenitor cells termed dental pulp stem cells (DPSCs). DPSCs can differentiate into odontoblast-like cells and maintain pulp homeostasis by the formation of new dentin which protects the underlying pulp. DPSCs similar to other mesenchymal stem cells (MSCs) reside in a niche, a complex microenvironment consisting of an extracellular matrix, other local cell types and biochemical stimuli that influence the decision between stem cell (SC) self-renewal and differentiation. In addition to biochemical factors, mechanical factors are increasingly recognized as key regulators in DPSC behavior and function. Thus, microenvironments can significantly influence the role and differentiation of DPSCs through a combination of factors which are biochemical, biomechanical and biophysical in nature. Under *in vitro* conditions, it has been shown that DPSCs are sensitive to different types of force, such as uniaxial mechanical stretch, cyclic tensile strain, pulsating fluid flow, low-intensity pulsed ultrasound as well as being responsive to biomechanical cues presented in the form of micro- and nano-scale surface topographies. To understand how DPSCs sense and respond to the mechanics of their microenvironments, it is essential to determine how these cells convert mechanical and physical stimuli into function, including lineage specification. This review therefore covers some aspects of DPSC mechanoresponsivity with an emphasis on the factors that influence their behavior. An in-depth understanding of the physical environment that influence DPSC fate is necessary to improve the outcome of their therapeutic application for tissue regeneration.

## Introduction

Dental pulp tissue contains an accessible source of multipotent mesenchymal progenitor cells, known as dental pulp stromal/stem cells (DPSCs), which participate in dentin and pulp regeneration (Gronthos et al., 2000). In the tooth, a specialized microenvironment called the stem cell niche exists and is located at specific anatomic sites which regulate how DPSCs participate in tissue homeostasis, regeneration and repair (Mitsiadis et al., 2011). The dental pulp is contained in the pulp cavity of the tooth within its hard tissues. The pulp is composed of four layers: an external layer containing the odontoblasts, a cell-free zone, a cell-rich zone and the pulp core. The external layer is comprised of odontoblasts produce the dentin extracellular matrix (ECM) that protects the pulp from external stimuli. The cell-free zone lacks cells and is rich in ECM, the cell-rich zone contains progenitor cells that exhibit plasticity and pluripotentiality whereas the central area contains the vasculature and nerve plexus (D’Aquino et al., 2008), these structures are also present at the periphery of the pulp in a close functional relationship with odontoblasts.

A key study by Lizier et al. (2012) indicated that DPSCs are located in multiple niches, which are associated with capillaries and the nerve network of the central region, in the cell rich-zone and in the outer layer. Importantly, there are specific interactions which occur between DPSCs and their local microenvironment in these niches (Mitsiadis et al., 2007, 2011) with DPSCs being functionally regulated by the local ECM, growth factors, other bioactive molecules and key signaling pathways (Mitsiadis et al., 2011). These factors act synergistically or antagonistically and as a network to regulate the status of DPSCs in the niches. Notably a complex network of biochemical signaling pathways including Notch and WNT/β-Catenin signaling, many growth factors such as vascular endothelial growth factor (VEGF), transforming growth factor (TGF)-β and ECM proteins participate in maintaining and regulating homeostasis in the DPSC niche as well as in the regulation of the proliferation and differentiation of DPSCs (Mitsiadis et al., 2007; Yu et al., 2015).

The teeth are essential for mastication and are subjected to various mechanical stresses due to jaw movement and occlusal forces which are transmitted to the dental pulp tissue and can subsequently influence DPSC fate (Smith et al., 2001; Cai et al., 2011; Hata et al., 2012). Moreover, odontoblasts and DPSCs contained within the dental pulp are responsible for dentin formation and its repair due to their ability to sense stress such as the biomechanical forces that occur during trauma (Kraft et al., 2011). Indeed, the dentin-pulp complex in response to external insults possesses the capacity to repair itself by producing dentin, in a process known as dentinogenesis (Loison-Robert et al., 2018). The dentin formed in response to such condition is called tertiary dentin, which may be deposited by either of two mechanisms known as reactionary and reparative dentinogenesis. Reactionary dentinogenesis occurs in response to mild tissue damage, whereby post-mitotic primary odontoblasts located at the periphery of the pulp secrete a tertiary dentin matrix in response. Reparative dentinogenesis occurs when the damage to the pulp is more substantive, resulting in the death of resident odontoblasts. As a consequence, there is recruitment of stem/progenitor cells that differentiate into new odontoblast-like cells which subsequently secrete a tertiary dentin matrix(Loison-Robert et al., 2018).

The signals involved in regulating SC fate are not only ECM proteins, adjacent differentiated cells, secreted and cell surface molecules but also mechanical signals. Notably it has been reported that in response to degradation, disruption and mechanical erosion DPSCs can differentiate into odontoblast-like cells to form dentin. Indeed several studies have now demonstrated that mechanical stresses transmitted to the pulp tissue can affect the behavior of DPSCs (Cai et al., 2011). Therefore, the formation and differentiation of odontoblast-like cells from DPSCs depend on signal transduction from the environment, including both chemical and physical cues (Tucker et al., 1998; Tatullo et al., 2016).

Dental pulp stem cells not only play a crucial role in dentinogenesis but also provide a promising source of cells for use in regenerative medicine (Hata et al., 2012). DPSCs can differentiate into many cell types including osteoblasts, neuronal cells and adipocytes (Mitsiadis et al., 2011; Marrelli et al., 2015). Several studies have also shown the potential of DPSCs for repair and regeneration of various tissues, such as teeth, bone, muscles, and heart (Gronthos et al., 2000; Mitsiadis et al., 2011). Importantly, DPSCs can be relatively easily harvested from a patient’s wisdom teeth [or even stem cells from human exfoliated deciduous teeth (SHED)], subsequently expanded, manipulated and returned to the same patient when tissue repair is required.

### Effects of Mechanical Stimuli on the Biological Behavior of DPSCs

**FIGURE 1:**
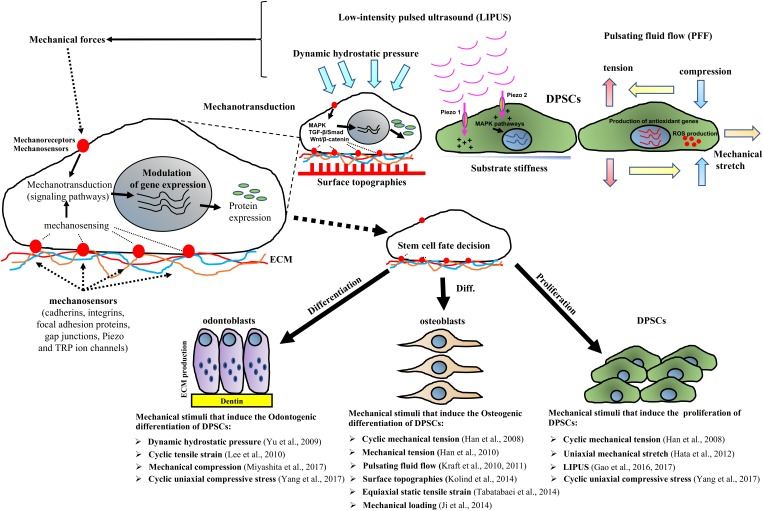
Schematic representation showing that mechanical factors stimulate stem cells through the activation of mechanosensors such as cadherins, integrins, focal adhesion proteins, gap junctions, cytoskeleton, Piezo and TRP ion channels, which subsequently trigger signaling pathways such as MAPK, TGF-β/Smad and Wnt/β-catenin cascades that modulate gene expression. Mechanical stimuli including mechanical stretch, cyclic tensile strain tension, compression, tension, pulsating fluid flow (PFF), low-intensity pulsed ultrasound (LIPUS) as well as surface topographies and substrate stiffness affect DPSCs responses such as by promoting DPSC proliferation and/or osteo/odontogenic differentiation. The control of the mechanical cues has application in DPSCs therapy approaches for tissue regeneration.

Sübay et al. (2001) have shown that orthodontic extrusive forces applied to the teeth did not cause significant pathological changes in human pulp tissue. However, due to the difficulty in studying the role of mechanical stimuli on DPSC behavior *in vivo*, many studies have investigated the effects of mechanical forces using *in vitro* models (Table 1). In this context, several studies have shown that mechanical stimuli including cyclic mechanical tension, low-intensity pulsed ultrasound (LIPUS), uniaxial mechanical stretch and cyclic uniaxial compressive stress are able to induce the proliferation of DPSCs (Han et al., 2008; Hata et al., 2012; Gao et al., 2016, 2017; Yang et al., 2017). Furthermore physical stimuli such as loading, surface topographies, dynamic hydrostatic pressure and pulsating fluid flow can reportedly promote the differentiation of DPSCs (see Table 1 and Figure 1) (Han et al., 2008, 2010; Yu et al., 2009; Kraft et al., 2010, 2011; Lee et al., 2010; Ji et al., 2014; Kolind et al., 2014; Tabatabaei et al., 2014; Miyashita et al., 2017; Yang et al., 2017). Several investigations have also shown that mechanical stimuli including dynamic hydrostatic pressure, cyclic tensile strain, mechanical compression and cyclic uniaxial compressive strain can promote the odontogenic differentiation of DPSCs (Yu et al., 2009; Lee et al., 2010; Miyashita et al., 2017; Yang et al., 2017). While other studies have demonstrated that cyclic mechanical tension, pulsating fluid flow, surface topographies, equiaxial static tensile strain and mechanical loading can promote the osteogenic differentiation of DPSCs (Han et al., 2008, 2010; Kraft et al., 2010, 2011; Ji et al., 2014; Kolind et al., 2014; Tabatabaei et al., 2014). Interestingly, mechanical forces such as uniaxial stretch can increase the proliferation of DPSCs while inhibiting the odontogenic and osteogenic differentiation of DPSCs, indicating that mechanical stimuli are therefore critical and contextually important in modifying DPSC fate (Cai et al., 2011; Hata et al., 2012).

**Table 1:** Studies identified between 2001 and 2017 that analyze the effects of mechanical stimuli on dental pulp stem cell behavior.

Mechanical stimulus	Stimulus description	Cell/Tissue description	Response	Reference
Mechanical tension (tensile strain, mechanical stretch)	Cyclic mechanical tension	Human DPSCs	Mechanical tension acted as a potent positive modulator of proliferation, osteogenic differentiation and ECM production in DPSCs.	Han et al., 2008
	Mechanical tension	Human DPSCs	Mechanical stimulation promotes osteogenesis in DPSCs	Han et al., 2010
	Cyclic tensile strain	Human Dental Pulp Cells (HDP)	Mechanical strain activates inflammatory cytokines and oxidative stress, which then act in concert to induce the Nrf2-/ARE-mediated antioxidant enzymes	Lee et al., 2008
	Cyclic tensile strain	Human dental pulp cells (HDPCs) immortalized with human telomerase transcriptase gene	MS stimulates odontoblastic differentiation of HDPCs via modulation of the Nrf2-mediated HO-1 pathway.	Lee et al., 2010
	Uniaxial cyclic tensile stretch	Human DPSCs	Cyclic tensile stretch inhibits the osteogenic and odontogenic differentiation of dental pulp stem cells	Cai et al., 2011
	Mechanical stretch	Human DPSCs	Dental pulp stem cells express tendon markers under mechanical loading	Chen et al., 2016
	Uniaxial mechanical stretch	Rat DPSCs	Uniaxial stretch increased the proliferation of DPSCs, while suppressing osteogenic differentiation. These results suggest a crucial role of mechanical stretch in the preservation of DPSCs in dentin	Hata et al., 2012
	Equiaxial static tensile strain	Human dental pulp stem cells (hDPSCs)	Static equiaxial strain which mimics the types of orthodontic forces can result in differentiation of hDPSCs to osteoblasts.	Tabatabaei et al., 2014
Mechanical loading	Mechanical loading	Human DPSCs	Dental pulp stem cells express tendon markers under mechanical loading	Chen et al., 2016
	Mechanical loading that mimic tooth-chewing movement	Human dental pulp stro- mal cells (hDPSCs)	Mechanical loading seems to promote the osteogenic potential for real bone-like matrix formation	Ji et al., 2014
	Mechanical loading in spinner flask bioreactors	Human dental pulp stem cells (hDPSCs) seeded on porous silk fibroin scaffolds	Mechanical loading is able to increase the mineralization potential of hDPSCs seeded on porous silk fibroin scaffolds	Woloszyk et al., 2014
Pulsating fluid flow (PFF)	Pulsating fluid flow (PFF)	Human DPSCs	DPSCs show a bone cell-like response to mechanical loading by PFF, and PDSC-mature show a more pronounced NO and PGE2 response to mechanical loading by PFF.	Kraft et al., 2010
	Pulsating fluid flow (PFF)	Human dental pulp-derived cells (DPC)	DPC show a bone cell-like response to mechanical load by PFF and DPC exposed to mineralizing conditions display more pronounced NO production than undifferentiated cells	Kraft et al., 2011
Micro and nanoscale surface topographies	Biomechanical cues presented in the form of micro and nanoscale surface topographies	Human mesenchymal dental pulp-derived stem cells (DPSCs)	Osteogenic inducers affect the influence of surface topography on DPSC differentiation along the osteogenic lineage	Kolind et al., 2014
	Mechanical influence of tissue culture plates and extracellular matrix	Human dental pulp stem cells (hDPSCs)	Mechanical and geometrical factors can influence DPSCs behavior and fate	Tatullo et al., 2016
Low-intensity pulsed ultrasound (LIPUS)	Low-intensity pulsed ultrasound (LIPUS)	Rat DPSCs	LIPUS promoted DPSCs proliferation in an intensity and cell-specific dependent manner via activation of distinct MAPK pathways	Gao et al., 2016
	Low-intensity pulsed ultrasound (LIPUS)	Rat DPSCs	This study demonstrated the presence of the membrane ion channels Piezo1 and Piezo2 in DPSCs. Piezo-dependent stimulation of ERK1/2 phosphorylation is involved in promoting DPSC proliferation after LIPUS treatment	Gao et al., 2017
Mechanical compression	Mechanical stress (compressive stress)	Human deciduous dental pulp stem cells (DDPSCs) and permanent dental pulp stem cells (DPSCs)	Expression levels of SLURP-1 and α7 nAChR in DPSCs increased with mechanical force stimulation. α7 nAChRs in DDPSCs were activated by SLURP-1 to up-regulate the expression of NF-κB and enhance its activity, which resulted in the promotion of osteoclastogenesis during the physiological root resorption of deciduous teeth.	Wang et al., 2017
	Mechanical compression	Human dental pulp stem cells (hDPSCs)	Odontoblastic differentiation of hDPSCs is promoted by optimal mechanical compression through the MAPK signaling pathway and expression of the BMP7 and Wnt10a genes	Miyashita et al., 2017
	Cyclic uniaxial compressive stress	Human dental pulp stem cells (hDPSCs)	Proliferation and odontogenic differentiation were significantly promoted in DPSCs subjected to cyclic uniaxial compressive stress	Yang et al., 2017
Mechanical forces that mimic parafunctional masticatory forces	Orthodontic extrusive force applications	Human Pulpal Tissue	Extrusive forces applied in this study did not cause significant pathological changes in human pulp tissue	Sübay et al., 2001
	Dynamic hydrostatic pressure (HSP) that simulate intra-pulpal pressure	Human DPSCs	HSP-treated DPSCs displayed enhanced odontogenic differentiation	Yu et al., 2009


Studies were categorized into groups according to the type of mechanical stimulus used.

The positive effects of mechanical force on DPSC proliferation and osteogenic/odontogenic differentiation are well described (Figure 1 and Table 1), however, a few researchers have reported that these forces have no significant effect on dental pulp cells (Sübay et al., 2001) or indeed have negative effects on the osteogenic and odontogenic differentiation of DPSCs (Cai et al., 2011; Hata et al., 2012).

Interestingly, it has also been shown that mechanical stimulation of deciduous pulp stem cells (SHEDs), promotes their osteoclastogenic differentiation which occurs during physiologic root resorption of deciduous teeth (Wang et al., 2017). Notably, using an *in vitro* model that mimicked the occlusal force during chewing movements, Wang et al. (2017) showed that the expression levels of the mammalian Ly-6 urokinase-type plasminogen activator receptor-associated protein 1 (SLURP-1) and alpha 7 nicotinic acetylcholine receptor (α7 nAChR) in deciduous DPSCs increased with mechanical stimulation. Subsequently, there was an activation of the NF-kB signaling and the promotion of osteoclastogenesis that ultimately resulted in root resorption (Wang et al., 2017).

The overall aim of this review therefore is to report the effects of mechanical stimuli on the biological behavior of DPSCs as well as describing the associated intracellular signaling and odonto/osteogenic differentiation in DPSCs. The data discussed in this review indicates that appropriate mechanical stresses are important biological stimuli that can effectively promote proliferation and differentiation of DPSCs. The understanding of how these cells respond to mechanical stimuli (mechanosensitivity) is important for bone and tooth tissue engineering applications using DPSCs alone or in conjunction with biomaterials and other bioactive molecules such as growth factors and cytokines.

## Dpsc Mechanosensing and Mechanical Stimuli that Induce Their Proliferation

Dental pulp stem cells are mechanosensitive cells that can recognize mechanical changes and transform this information into cellular responses (Kraft et al., 2011). For example, it has been shown that low-intensity pulsed ultrasound (LIPUS), a potential therapy for dental tissue repair, can stimulate mitogen-activated protein kinase (MAPK) signaling and induce the proliferation of DPSCs (Gao et al., 2016). In this context, several cell membrane proteins such as ion channels are implicated in the mechanosensing mechanism (Gao et al., 2017). Indeed Gao et al. (2017) observed that Piezo-1 and -2, two transmembrane cation channels, are important cell membrane–located mechanotransduction components expressed on DPSCs and involved in activating intracellular signaling that supports cellular responses. The authors observed that the levels of Piezo-1 and -2 proteins in DPSCs were increased after LIPUS treatment and that the LIPUS-mediated stimulation of DPSC proliferation was subsequently mediated by ERK1/2 MAPK signaling (Gao et al., 2017). These findings indicate that the Piezo ion channels present on DPSC membranes are able to transduce the LIPUS stimuli into a biological response.

Three other studies have shown that mechanical stimuli including cyclic mechanical tension, uniaxial mechanical stretch and cyclic uniaxial compressive stress can significantly increase DPSC proliferation *in vitro* (Table 1 and Figure 1) (Han et al., 2008; Hata et al., 2012; Yang et al., 2017). In contrast, Yu et al. (2009) reported that mechanical forces that mimic parafunctional masticatory forces *in vivo* were able to reduce DPSC survival and adhesion through the downregulation of the adhesion markers ICAM-1 and VCAM-1. These authors concluded that additional studies were required to clarify the role of intra- and inter-cellular adhesion molecules and other cellular pathways associated with DPSCs subjected to cellular stress. The process whereby cells translate mechanical inputs into biochemical signals known as mechanotransduction has been well described in the literature (D’Angelo et al., 2011). Briefly, the mechanotransduction occurs through numerous mechanoreceptors and mechanosensors, including cadherins, integrins, focal adhesion proteins, gap junctions, cytoskeleton and ion channels such as Piezo and transient receptor potential (TRP) ion channels, which can modify the gene expression levels in response to mechanical stimuli by activating downstream signaling pathways (Pavalko et al., 2003; Papachristou et al., 2009; Yin and Kuebler, 2010; Steward et al., 2014; Gao et al., 2017). Several classical pathways are able to transduce mechanical stimuli to biochemical signals including the mitogen-activated protein kinases (p38 MAPK, ERK, and JNK) or TGF-β/Smad and Wnt/β-catenin signaling cascade. These biochemical cascades are linked to transcription factor activation and thus to the expression of genes which are crucial for stem cell-fate decision (Figure 1). In this context, it is also important to stress that the changes in the expression of transcription factors are influenced not only by mechanical stimuli but also by the microenvironment in which the stem cells reside. Therefore, synchronized interactions with the neighboring cells, soluble factors and ECM produce mechanical and biochemical signals that enable the stem cells to survive, proliferate or differentiate (Li et al., 2011).

## Mechanical Stimuli that Induce Osteogenic Differentiation of DPSCs

A recent study by Han et al. (2008) investigated the effects of mechanical tension on DPSC proliferation and differentiation. They observed that a cyclic mechanical tension (8% strain) increased the proliferation and mRNA expression levels of the osteogenic markers osteopontin and type I collagen and decreased the mRNA expression levels of smooth muscle actin (α-SMA) and the surface protein CD90 (Han et al., 2008). These findings suggest that mechanical cyclic tension can be considered a potent positive modulator of osteogenic differentiation by DPSCs. Similarly, the same authors showed that cyclic mechanical stretch increased the transcript levels of collagen I, fibronectin and osteoprotegerin in these cells whereas it decreased the expression levels of α-SMA. Moreover, other osteogenic-related proteins including collagen I, bone sialoprotein (BSP), osteocalcin and osteonectin increased in DPSCs that were subjected to mechanical cyclic tension (Han et al., 2010). These data suggest that specific mechanical stimuli such as stretch can effectively enhance osteogenic differentiation of DPSCs.

*In vitro* mechanical stimuli including pulsating fluid flow (PFF), equiaxial static tensile strain and micro- and nano-scale surface topographies have also been shown to induce osteogenic differentiation of DPSCs (Kraft et al., 2010; Kolind et al., 2014; Tabatabaei et al., 2014). For example, DPSCs in a similar manner to osteocytes (Kleinnulend et al., 1995) are able to respond to PFF, which is used to mimic the mechanical loading on the dental pulp under *in vivo* conditions. Subsequently DPSCs responded to PFF by increasing the production of markers that were positively correlated with increased mechanoresponsiveness of osteocytes after mechanical loading such as nitric oxide (NO), prostaglandin E_2_ (PGE_2_) and cyclooxygenase-2 (COX-2) (Kraft et al., 2010, 2011).

As has been observed for osteocytes, the rapid stimulation of NO production by PFF in DPSCs appears to be related to the activity of the enzyme endothelial nitric oxide synthase (eNOS) but not to the activity of inducible NOS (iNOS) (Kraft et al., 2011). Importantly, by comparing the response to PFF in two DPSC lines, both with osteogenic potential, one immature and the other more mature, it was shown that the more mature cells possessed a more osteocytic mechanoresponsiveness compared with the immature DPSCs. As has been shown for osteocytes, these data indicated that the osteogenic maturation of DPSCs was influenced by their mechanoresponsivity, and there was a positive correlation between the degree of osteogenic maturation and increased mechanical stimulation (Kraft et al., 2010).

An interesting study by Tabatabaei et al. (2014) investigated whether equiaxial mechanical strain could be used to induce the osteogenic differentiation of DPSCs in the absence of other osteogenic induction agents. Interestingly, they observed that a mechanical stimulus that mimicked the orthodontic forces, such as static equiaxial strain, increased the expression levels of the osteogenic markers osteopontin and alkaline phosphatase (ALP), and therefore reportedly effectively induced the differentiation of human DPSCs into osteoblasts(Tabatabaei et al., 2014).

Furthermore, using a bioreactor that mimicked biting force, Ji et al. (2014) successfully developed a novel method to enhance the osteogenic differentiation of DPSCs. They showed that the application of mechanical forces that mimicked the dynamics of those exerted *in vivo* on DPSCs could be used to promote bone formation and limit bone resorption (Ji et al., 2014).

A further study by Woloszyk et al. (2014) in the same year evaluated the mineralization potential of human DPSCs seeded on porous silk fibroin scaffolds in a mechanically dynamic environment established using spinner flask bioreactors. Interestingly, these authors showed that DPSCs are reactive to mechanical loading, which also affects bone and that the mechanical loading increased the mineralization of silk scaffolds seeded with DPSCs. Therefore, these authors demonstrated that loading in the form of turbulent flow can accelerate the process of mineral deposition of DPSCs (Woloszyk et al., 2014).

## Mechanical Stimuli that Induce the Odontogenic Differentiation of DPSCs

It has previously been shown that abnormal occlusal forces generated from malocclusion or orthodontic appliances can induce abnormal mineralization such as root canal calcification and pulp stone formation as well as lead to cell death and root absorption (Landay et al., 1970; Yu et al., 2009). Moreover, chronic parafunctional forces such as those derived from bruxism and clenching can generate an elevated intra-pulpal pressure, pulpal inflammation and the formation of calcified nodules(Yu et al., 2009).

The formation of mineralized tissue in response to mechanical forces that mimic the parafunctional masticatory forces has been studied by Yu et al. (2009). They showed that the dynamic hydrostatic pressure (HSP) that mimics the sustained intra-pulpal pressure in *in vivo* conditions can induce odontogenic differentiation as well as mineralization in DPSC cultures *in vitro* (Yu et al., 2009). More importantly, DPSCs that were subjected to HSP seeded within a bed of hydroxyapatite/tricalcium phosphate (HA-TCP) were found to be more responsive to the stimulatory effects of BMP-2 *in vivo*. The authors also reported that these HSP-induced DPSCs can induce the formation of hard tissue.

Several other *in vivo* studies have been performed which demonstrate the response of dental pulp tissue to mechanical forces (Shibutani et al., 2010; Yang et al., 2017). Interestingly, it has been observed that excessive occlusal forces can induce changes in mineral deposition and in the microvasculature structure in the dental pulp tissue (Shibutani et al., 2010). To better understand the effect of mechanical stress on DPSCs differentiation, Yang et al. (2017) showed that compressive stress was able to induce several changes in DPSCs, including proliferation, cell morphology and odontogenic differentiation. DPSCs under compressive stress increased their expression of the odontogenic-related transcripts of *ALP*, *DMP1*, *BMP2*, *DSPP* and *COL I*, and therefore subsequently concluded that mechanical stimuli could be used to initiate repair mechanisms within the dentin-pulp complex (Yang et al., 2017).

A recent study by Miyashita et al. (2017) demonstrated a novel mechanism of mechanical induction of odontoblastic differentiation of DPSCs. Their research showed that mechanical compression promoted differentiation of these cells through the MAPK, ERK1/2 and p38 signaling pathways, as well as through the expression of the bone morphogenetic protein 7 (BMP-7) and the wingless-type MMTV integration site family member 10a (Wnt10a) (Miyashita et al., 2017).

## Mechanical Stimuli that Induce the Activation of Proinflammatory Cytokines and Antioxidant Defense Enzyme Release in DPSCs

The biological response of DPSCs to mechanical stimuli occurs not only during orthodontic tooth movement but also during normal mastication. For example, DPSCs can produce growth factors, angiogenic changes and a mild inflammatory-type reaction in response to orthodontic forces (Lee et al., 2008, 2010). Furthermore it is notable that there is activation of pro-inflammatory cytokines and antioxidant defense enzymes in DPSCs subjected to physiological mechanical stress (Lee et al., 2008). Lee et al. (2008) have shown that the *in vitro* mechanical loading of DPSCs by cyclic strain stimulates the production of inflammatory cytokines including interleukins (ILs)-6 and -1β, tumour necrosis factor (TNF)-α as well as the expression of the antioxidant genes heme oxygenase-1 (HO-1) and superoxide dismutases (SOD). The same authors also showed that mechanical stimulation using cyclic tensile strain induced expression of the odontoblastic markers DSPP, DMP-1, OPN, and BSP. Moreover, they demonstrated that the odontoblastic differentiation of DPSCs was mediated by the NF-E2-related transcription factor 2 (Nrf2)/HO-1 pathway (Lee et al., 2010). These data suggest that it is possible to target the HO-1 pathway to manipulate odontogenic differentiation of DPSCs subjected to mechanical stress. Further studies also indicated that a limited amount of mechanical stress was appropriate to stimulate physiological metabolism and to induce odontoblastic differentiation of DPSCs, and subsequently the formation of tertiary dentin (Lee et al., 2008). In support of this, clinical studies have demonstrated that mechanical biting of teeth during mastication can produce a relatively thick dentin, whereas impacted teeth did not produce the same type of dentin as they are not used during mastication and therefore not exposed to mechanical stress (Mjör, 2002).

## Conclusion and Future Prospective

Dental pulp tissue is subjected to mechanical stress during normal masticatory process and also during pathological trauma or orthodontic tooth movement (Lee et al., 2010; Yang et al., 2017).

Moreover, it is also well known that occlusive force plays a role in physiological root resorption of deciduous teeth, in which dental pulp cells from these mechanically stressed teeth secrete cytokines that are important for odontoclast activation and subsequent root resorption (Lin et al., 2012; Wang et al., 2017).

Therefore, DPSCs located near the roots of teeth are subjected to higher levels of oral mechanical stress by jaw movement and occlusal forces. Moreover, orthodontic forces transfer horizontal stretch to DPSCs, and also during tooth eruption dental pulp tissue is stretched vertically (Hata et al., 2012).

Furthermore, during physiological mechanical loading of teeth, the dentin is subjected to fluid flow which can activate the nocireceptors and mechanoreceptors on odontoblast processes present in the dentin tubules that regulate the maintenance of tooth integrity (Paphangkorakit and Osborn, 2000).

Importantly, Kraft et al. (2010, 2011) in two separate studies, found that the mechanism that govern the DPSCs response to mechanical loading is similar to that observed for osteocytes. In fact, they observed that the odontoblastic progenitors present in dental pulp are able to repair and form dentin by sensing mechanical stimuli such as PFF through NO production. NO is well known to play a key role in the response of bone to mechanical loading, indicating that DPSCs exhibit a similar response to this mechanical stimulus (Kolind et al., 2014). Therefore, this mechanoresponsiveness of DPSCs suggests that these cells can be used for bone tissue engineering applications such as the repair of maxillofacial defects.

To the best of our knowledge, until now only the study by Gao et al. (2017) has demonstrated that DPSCs sense mechanical stimuli by using the membrane ion channels of Piezo-1 and -2, interesting other authors are yet to investigate how DPSCs sense mechanical forces.

Regenerative medicine is an important application of DPSCs and this necessitates a thorough knowledge of mechanobiology. However, the studies analyzed in this review demonstrate that the response of DSPCs to mechanical stimuli differs according to the type and source of the mechanical forced applied (Table 1 and Figure 1). Importantly, we would like to underline that the literature reviewed in Table 1 contains studies with different approaches and experimental conditions. Therefore, it is very difficult to determine which approach may represent the “best” mechanical stimulus able to induce tissue regeneration or proliferation. We hypothesize that, among them, low-intensity pulsed ultrasound (LIPUS) is one of the most promising mechanical stimuli that could be used for future clinical applications as it is regarded as an economical, relatively straightforward and safe therapeutic approach as well as having reported ability to enhance the viability, proliferation and multilineage differentiation of several types of MSCs(Gao et al., 2016, 2017).

The understanding of the mechanical stimuli that regulate DPSC behavior will not only improve our knowledge relating to mechanisms involved in their differentiation but could also provide valuable insights for optimizing DPSC-based therapies. In fact, the particular mechanoresponsiveness of DPSCs to mechanical stimuli may be of utility for future bone and teeth tissue engineering applications.

Although physical and mechanical factors are known to play a key role in regulating DPSC fate, further studies are required to elucidate the detailed molecular mechanisms and signaling pathways involved in the mechanosensitive response of DPSCs to various types of forces.Ultimately it may be possible to use mechanical forces that mimic the dynamics of the *in vivo* environment on DPSCs to promote the regeneration of dental and bone tissues.

## Author Contributions

FP, MM, BC, RS, BS, PC, and MT conceived and wrote the manuscript. All authors reviewed and approved the final manuscript.

## Conflict of Interest Statement

The authors declare that the research was conducted in the absence of any commercial or financial relationships that could be construed as a potential conflict of interest.

## References

[B1] Cai X. Zhang Y. Yang X. Grottkau B. E. Lin Y. (2011). Uniaxial cyclic tensile stretch inhibits osteogenic and odontogenic differentiation of human dental pulp stem cells. *J. Tissue Eng. Regen. Med.* 5 347–353. 10.1002/term.319 20827678

[B2] Chen Y.-Y. He S.-T. Yan F.-H. Zhou P.-F. Luo K. Zhang Y.-D. (2016). Dental pulp stem cells express tendon markers under mechanical loading and are a potential cell source for tissue engineering of tendon-like tissue. *Int. J. Oral Sci.* 8:213. 10.1038/ijos.2016.33 27811845 PMC5168414

[B3] D’Angelo F. Tiribuzi R. Armentano I. Kenny J. M. Martino S. Orlacchio A. (2011). Mechanotransduction: tuning stem cells fate. *J. Funct. Biomater.* 2 67–87. 10.3390/jfb2020067 24956164 PMC4030896

[B4] D’Aquino R. Papaccio G. Laino G. Graziano A. (2008). Dental pulp stem cells: a promising tool for bone regeneration. *Stem Cell Rev.* 4 21–26. 10.1007/s12015-008-9013-5 18300003

[B5] Gao Q. Cooper P. R. Walmsley A. D. Scheven B. A. (2017). Role of piezo channels in ultrasound-stimulated dental stem cells. *J. Endod.* 43 1130–1136. 10.1016/j.joen.2017.02.022 28527849

[B6] Gao Q. Walmsley A. D. Cooper P. R. Scheven B. A. (2016). Ultrasound stimulation of different dental stem cell populations: role of mitogen-activated protein kinase signaling. *J. Endod.* 42 425–431. 10.1016/j.joen.2015.12.019 26830427

[B7] Gronthos S. Mankani M. Brahim J. Robey P. G. Shi S. (2000). Postnatal human dental pulp stem cells (DPSCs) *in vitro* and *in vivo*. *Proc. Natl. Acad. Sci. U.S.A.* 97 13625–13630. 10.1073/pnas.240309797 11087820 PMC17626

[B8] Han M.-J. Seo Y.-K. Yoon H.-H. Song K.-Y. Park J.-K. (2008). Effect of mechanical tension on the human dental pulp cells. *Biotechnol. Bioprocess Eng.* 13 410–417. 10.1007/s12257-008-0146-9 20143021

[B9] Han M.-J. Seo Y.-K. Yoon H.-H. Song K.-Y. Park J.-K. (2010). Upregulation of bone-like extracellular matrix expression in human dental pulp stem cells by mechanical strain. *Biotechnol. Bioprocess Eng.* 15 572–579. 10.1007/s12257-009-0102-3

[B10] Hata M. Naruse K. Ozawa S. Kobayashi Y. Nakamura N. Kojima N. (2012). Mechanical stretch increases the proliferation while inhibiting the osteogenic differentiation in dental pulp stem cells. *Tissue Eng. Part A* 19 625–633. 10.1089/ten.tea.2012.0099 23153222 PMC3566654

[B11] Ji J. Sun W. Wang W. Munyombwe T. Yang X. B. (2014). The effect of mechanical loading on osteogenesis of human dental pulp stromal cells in a novel in vitro model. *Cell Tissue Res.* 358 123–133. 10.1007/s00441-014-1907-8 24916612

[B12] Kleinnulend J. Semeins C. Ajubi N. Nijweide P. Burger E. (1995). Pulsating fluid flow increases nitric oxide (NO) synthesis by osteocytes but not periosteal fibroblasts-correlation with prostaglandin upregulation. *Biochem. Biophys. Res. Commun.* 217 640–648. 10.1006/bbrc.1995.2822 7503746

[B13] Kolind K. Kraft D. Boggild T. Duch M. Lovmand J. Pedersen F. S. (2014). Control of proliferation and osteogenic differentiation of human dental-pulp-derived stem cells by distinct surface structures. *Acta Biomater.* 10 641–650. 10.1016/j.actbio.2013.11.006 24252446

[B14] Kraft D. C. Bindslev D. A. Melsen B. Abdallah B. M. Kassem M. Klein-Nulend J. (2010). Mechanosensitivity of dental pulp stem cells is related to their osteogenic maturity. *Eur. J. Oral Sci.* 118 29–38. 10.1111/j.1600-0722.2009.00709.x 20156262

[B15] Kraft D. C. E. Bindslev D. A. Melsen B. Klein-Nulend J. (2011). Human dental pulp cells exhibit bone cell-like responsiveness to fluid shear stress. *Cytotherapy* 13 214–226. 10.3109/14653249.2010.487897 20491534

[B16] Landay M. A. Nazimov H. Seltzer S. (1970). The effects of excessive occlusal force on the pulp. *J. Periodontol.* 41 3–11. 10.1902/jop.1970.41.1.3 5264375

[B17] Lee S. K. Lee C. Y. Kook Y. A. Lee S. K. Kim E. C. (2010). Mechanical stress promotes odontoblastic differentiation via the heme oxygenase-1 pathway in human dental pulp cell line. *Life Sci.* 86 107–114. 10.1016/j.lfs.2009.11.013 19951713

[B18] Lee S.-K. Min K.-S. Jeong G.-S. Lee S.-H. Lee H.-J. Lee S.-I. (2008). Mechanical stress activates proinflammatory cytokines and antioxidant defense enzymes in human dental pulp cells. *J. Endod.* 34 1364–1369. 10.1016/j.joen.2008.08.024 18928848

[B19] Li D. Zhou J. Chowdhury F. Cheng J. Wang N. Wang F. (2011). Role of mechanical factors in fate decisions of stem cells. *Regen. Med.* 6 229–240. 10.2217/rme.11.2 21391856 PMC3128460

[B20] Lin B. C. Zhao Y. M. Yang J. Ge L. H. (2012). Root resorption of primary molars without successor teeth. An experimental study in the beagle dog. *Eur. J. Oral Sci.* 120 147–152. 10.1111/j.1600-0722.2012.00950.x 22409221

[B21] Lizier N. F. Kerkis A. Gomes C. M. Hebling J. Oliveira C. F. Caplan A. I. (2012). Scaling-up of dental pulp stem cells isolated from multiple niches. *PLoS One* 7:e39885. 10.1371/journal.pone.0039885 22768154 PMC3387222

[B22] Loison-Robert L. S. Tassin M. Bonte E. Berbar T. Isaac J. Berdal A. (2018). In vitro effects of two silicate-based materials, biodentine and bioroot rcs, on dental pulp stem cells in models of reactionary and reparative dentinogenesis. *PLoS One* 13:e0190014. 10.1371/journal.pone.0190014 29370163 PMC5784909

[B23] Marrelli M. Paduano F. Tatullo M. (2015). Human periapical cyst–mesenchymal stem cells differentiate into neuronal cells. *J. Dent. Res.* 94 843–852. 10.1177/0022034515570316 25672890

[B24] Mitsiadis T. Feki A. Papaccio G. Catón J. (2011). Dental pulp stem cells, niches, and notch signaling in tooth injury. *Adv. Dent. Res.* 23 275–279. 10.1177/0022034511405386 21677078

[B25] Mitsiadis T. A. Barrandon O. Rochat A. Barrandon Y. De Bari C. (2007). Stem cell niches in mammals. *Exp. Cell Res.* 313 3377–3385. 10.1016/j.yexcr.2007.07.027 17764674

[B26] Miyashita S. Ahmed N. E. M. B. Murakami M. Iohara K. Yamamoto T. Horibe H. (2017). Mechanical forces induce odontoblastic differentiation of mesenchymal stem cells on three-dimensional biomimetic scaffolds. *J. Tissue Eng. Regen. Med.* 11 434–446. 10.1002/term.1928 24920062

[B27] Mjör I. A. (2002). *Pulp-Dentin Biology In Restorative Dentistry.* Chicago: Quintessence. 11890026

[B28] Papachristou D. J. Papachroni K. K. Basdra E. K. Papavassiliou A. G. (2009). Signaling networks and transcription factors regulating mechanotransduction in bone. *Bioessays* 31 794–804. 10.1002/bies.200800223 19444851

[B29] Paphangkorakit J. Osborn J. (2000). The effect of normal occlusal forces on fluid movement through human dentine *in vitro*. *Arch. Oral Biol.* 45 1033–1041. 10.1016/S0003-9969(00)00090-X 11084142

[B30] Pavalko F. M. Norvell S. M. Burr D. B. Turner C. H. Duncan R. L. Bidwell J. P. (2003). A model for mechanotransduction in bone cells: the load-bearing mechanosomes. *J. Cell. Biochem.* 88 104–112. 10.1002/jcb.10284 12461779

[B31] Shibutani N. Hosomichi J. Ishida Y. Soma K. (2010). Influence of occlusal stimuli on the microvasculature in rat dental pulp. *Angle Orthod.* 80 316–321. 10.2319/012909-58.1 19905857 PMC8973234

[B32] Smith A. Tobias R. Murray P. (2001). Transdentinal stimulation of reactionary dentinogenesis in ferrets by dentine matrix components. *J. Dent.* 29 341–346. 10.1016/S0300-5712(01)00020-3 11472806

[B33] Steward A. J. Wagner D. R. Kelly D. J. (2014). Exploring the roles of integrin binding and cytoskeletal reorganization during mesenchymal stem cell mechanotransduction in soft and stiff hydrogels subjected to dynamic compression. *J. Mech. Behav. Biomed. Mater.* 38 174–182. 10.1016/j.jmbbm.2013.07.020 24054946

[B34] Sübay R. K. Kaya H. Tarım B. Sübay A. Cox C. F. (2001). Response of human pulpal tissue to orthodontic extrusive applications. *J. Endod.* 27 508–511. 10.1097/00004770-200108000-00003 11501587

[B35] Tabatabaei F. Jazayeri M. Ghahari P. Haghighipour N. (2014). Effects of equiaxial strain on the differentiation of dental pulp stem cells without using biochemical reagents. *Mol. Cell Biomech.* 11 209–220. 25831861

[B36] Tatullo M. Marrelli M. Falisi G. Rastelli C. Palmieri F. Gargari M. (2016). *Mechanical Influence of Tissue Culture Plates and Extracellular Matrix on Mesenchymal Stem Cell Behavior: A Topical Review.* London: SAGE Publications. 10.1177/0394632015617951 PMC5806742 26612837

[B37] Tucker A. S. Matthews K. L. Sharpe P. T. (1998). Transformation of tooth type induced by inhibition of BMP signaling. *Science* 282 1136–1138. 10.1126/science.282.5391.1136 9804553

[B38] Wang L. Zhou Z. Chen Y. Yuan S. Du Y. Ju X. (2017). The Alpha 7 nicotinic acetylcholine receptor of deciduous dental pulp stem cells regulates osteoclastogenesis during physiological root resorption. *Stem Cells Dev.* 26 1186–1198. 10.1089/scd.2017.0033 28494644

[B39] Woloszyk A. Holsten Dircksen S. Bostanci N. Muller R. Hofmann S. Mitsiadis T. A. (2014). Influence of the mechanical environment on the engineering of mineralised tissues using human dental pulp stem cells and silk fibroin scaffolds. *PLoS One* 9:e111010. 10.1371/journal.pone.0111010 25354351 PMC4213001

[B40] Yang H. Shu Y.-X. Wang L.-Y. Zou W.-L. Guo L.-Y. Shao M.-Y. (2017). Effect of cyclic uniaxial compressive stress on human dental pulp cells *in vitro*. *Connect. Tissue Res.* 59 255–262. 10.1080/03008207.2017.1367773 28816569

[B41] Yin J. Kuebler W. M. (2010). Mechanotransduction by TRP channels: general concepts and specific role in the vasculature. *Cell Biochem. Biophys.* 56 1–18. 10.1007/s12013-009-9067-2 19842065

[B42] Yu J. Jamal M. Garcia-Godoy F. Huang G. T.-J. (2015). “Dental pulp stem cell niche,” in *Tissue-Specific Stem Cell Niche* ed. Turksen K. (Berlin: Springer) 163–189. 10.1007/978-3-319-21705-5_8

[B43] Yu V. Damek-Poprawa M. Nicoll S. Akintoye S. (2009). Dynamic hydrostatic pressure promotes differentiation of human dental pulp stem cells. *Biochem. Biophys. Res. Commun.* 386 661–665. 10.1016/j.bbrc.2009.06.106 19555657 PMC2750776

